# Fate tracing of hepatocytes in mouse liver

**DOI:** 10.1038/s41598-017-15973-7

**Published:** 2017-11-23

**Authors:** Xiaowen Gu, Danyi Huang, Lei Ci, Jiahao Shi, Mengjie Zhang, Hua Yang, Zhugang Wang, Zhejin Sheng, Ruilin Sun, Jian Fei

**Affiliations:** 1 School of Life Science and Techonology, Tongji University, Shanghai, 200092 China; 2 Shanghai Engineering Research Center for Model Organisms, SRCMO/SMOC, Shanghai, 201203 China

## Abstract

Hepatocytes perform most of the functions of the liver and are considered terminally differentiated cells. Recently, it has been suggested that hepatocytes might have the potential to transdifferentiate or dedifferentiate under physiological or pathological conditions *in vivo*. Epithelial-mesenchymal transition of hepatocytes in liver fibrosis has also been proposed. However, these findings have not been fully confirmed. In this study, hepatocytes were genetically labelled for cell fate tracing using lacZ via the tamoxifen-induced CreERT/loxP system. After induction with tamoxifen, alb + cells were permanently marked by lacZ expression, and all progeny lacZ + cells were derived from a single source with no interference. We did not observe transdifferentiation or dedifferentiation of hepatocytes into cholangiocytes or hepatic progenitor cells under conditions of liver homeostasis or following a 2/3 partial hepatectomy. Meanwhile, lacZ/OPN-positive cells were observed in livers of 3,5-diethoxycarbonyl-1,4-dihydrocollidine-fed mice, and lacZ/alpha-smooth muscle actin-positive cells were detected in carbon tetrachloride-induced chronic liver injury models. These results suggested that some existing differentiated alb + cells might have the potential of transdifferentiation/dedifferentiation or epithelial-to-mesenchymal transition *in vivo* in some liver injury models, but the proportion of these alb + cells in liver was very low, and their significance and actual function during the pathological process remains to be elucidated.

## Introduction

The liver is considered unique among adult mammalian organs because of its incredible regenerative capability. Recent studies suggested that different liver cell types including hepatocytes, cholangiocytes, hepatic progenitor cells, and hepatic stellate cells (HSCs), have different roles in response to various types of liver injury. These liver cells might provide progeny cells during liver regeneration. Hepatocytes, cells of the main parenchymal tissue in the liver, perform most of the functions of the liver and have the capability to proliferate. When the liver undergoes a physical injury such as a partial hepatectomy, hepatocytes begin to proliferate rapidly to restore the weight and function of the liver^1^. Up to 75% of the liver mass can be regenerated within 1 week after a partial hepatectomy^2^. In animals with chemical injuries, such as a 3,5-diethoxycarbonyl-1,4-dihydrocollidine (DDC)-fed mouse model, hepatocytes rarely proliferate. Instead, it is suggested that cholangiocytes or oval cells are activated^3–5^. In a carbon tetrachloride (CCl4)-induced liver injury model, hepatocytes are considered ‘accomplices’ in progressive fibrosis by expressing TGF-β and fibromodulin, which promote fibrosis *in vivo* ^6,7^. It remains unclear whether hepatocytes generate their progeny cells via direct proliferation or whether they require a process of dedifferentiation into progenitor cells. In a previous study, mature hepatocytes were observed to express the progenitor cell markers CD45 and LMO2 after they were cultured *in vitro* for 15 days^8^. Acute inactivation of the Hippo signalling pathway *in vivo* was also considered sufficient to dedifferentiate adult hepatocytes into cells bearing progenitor characteristics *in vivo* ^9^. These studies suggested that hepatocytes might have the potential of transdifferentiate or dedifferentiate under certain conditions. However, Malato Y *et al*. studied the phenotypes of AAV-ttr system-labelled hepatocytes inseveral liver injury models and found no evidence that biliary injury induced the conversion of hepatocytes into cholangiocytes^10^. Recent studies have shown that hepatocytes actively contribute to liver fibrosis via expression of specific proteins involved in progressive liver fibrosis^11^. Adult mouse hepatocytes induced by TGF-β1 were found to undergo phenotypic and functional changes that are typical of epithelial-to-mesenchymal transition (EMT) *in vitro* ^12^. However, other researchers did not observe the EMT of hepatocytes *in vivo* ^13^. Therefore, whether hepatocytes undergo the EMT process remains unclear.

In this study, we used the albumin (Alb)-CreERT/Rosa26-LsL-LacZ system to trace hepatocytes and their progeny cells during liver homeostasis or indifferent liver repair processes *in vivo* via the lacZ tag. In this way, hepatocytes and their progeny cells avoided the additional stimulation caused by *in vitro* cell culture and they were not confused with hepatocytes of other origins.

Our results indicated that hepatocytes rarely showed their potential for transdifferentiation or dedifferentiation during liver homeostasis or during liver regeneration after physical liver injury. In contrast, lacZ/OPN-positive cells were observed in mice after a DDC diet, which indicated the transdifferentiation potential of hepatocytes. Additionally, in a CCl4-induced chronic liver injury model, lacZ + /alpha-smooth muscle actin (α-sma) + cells were detected, suggesting the EMT transition of hepatocytes. However, the proportion of these hepatocyte-derived cells in liver was very low, and their significance and actual function during the pathological process remains to be elucidated.

## Results

### Genetic labelling of hepatocytes and lineage tracing

**Figure 1:**
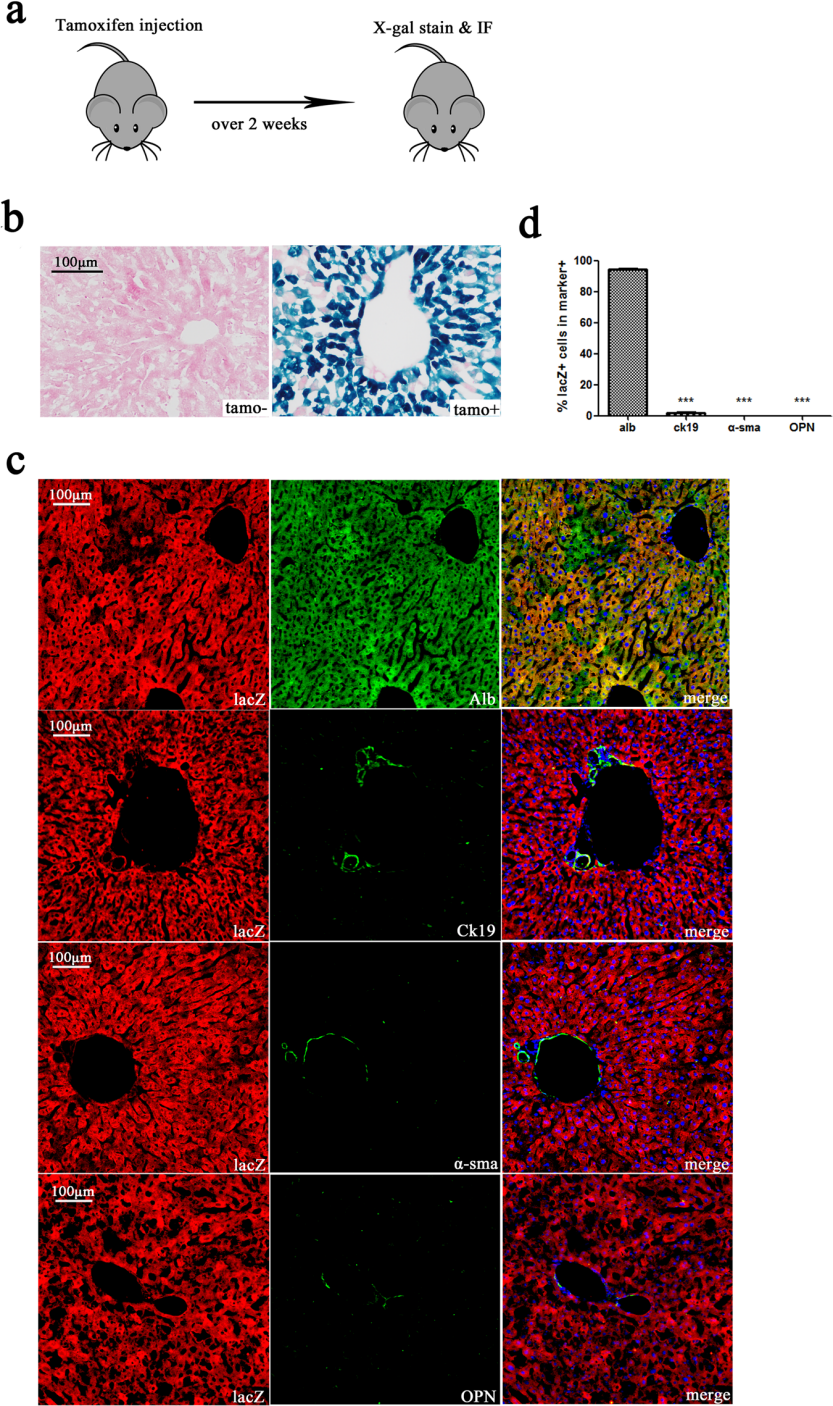
Tamoxifen-induced lacZ staining of hepatocytes. (**a**) Experimental scheme: Alb-lacZ-cre mice were injected with tamoxifen (120 mg/kg). After a 2-week washout period, mice were sacrificed. (**b**) Hepatocytes from Alb-cre-lacZ mice injected with tamoxifen were X-gal-positive, while those in the control group (injected with corn oil) were X-gal-stain negative. (**c**) Double immunostaining for lacZ and the hepatocyte marker albumin, the cholangiocytes marker Ck19, the HSC marker α-sma, or the liver stem cell marker OPN. (**d**) The ratio of lacZ + /marker + cells in marker + cells. The ratio of lacZ + /alb + cells in alb + cells (94.31% ± 2.81%) was significantly higher than that of in Ck19 + (1.76% ± 1.25%), α-sma (0.00% ± 0.00%), or OPN (0.00% ± 0.00%) cells. Nuclei were stained using DAPI (blue). Over 15 sections from a group of mice (n = 3) were counted; data are expressed as the mean ± SEM; magnification, × 200; ***p < 0.01.

To trace the lineage of mouse hepatocytes *in vivo*, we constructed an Alb-creERT mouse model (Supplementary Fig. s1a) using homologous recombination technology. Cre recombinase activity was controlled by tamoxifen, and cre recombinase expression was restricted to Alb + cells. Alb-creERT mice were confirmed to have normal liver function (Supplementary Fig. s1b). Alb-creERT mice were mated with Rosa26-LSL-lacZ mice to produce Alb-cre-lacZ mice for subsequent experiments. Adult Alb-cre-lacZ mice were divided into two group (n = 3 per group). Animals in the tamoxifen group received tamoxifen (120 mg/kg in corn oil) by intraperitoneal injection 5 times every second day, while the control group was injected with corn oil. Mice were sacrificed after being fed a normal diet for over 2 weeks to wash out tamoxifen (Fig. 1a). X-gal-positive stained cells were found in mouse livers of the tamoxifen group, but not in the control group, which indicated that lacZ expression was only activated after tamoxifen induction (Fig. 1b). The percentage of the liver area that was positive for X-gal staining (approximately 65.17% ± 6.40%) was measured to assess the percentage of lacZ + cells in the liver (Supplementary Fig. s1c). Livers were double immunostained for lacZ and the hepatocyte marker albumin (Alb), the cholangiocyte marker cytokeratin 19 (Ck19), the hepatic stellate cell (HSC) marker alpha-smooth muscle actin (α-sma), or the stem cell marker osteopontin (OPN). The results showed that hepatocytes were specially labelled with lacZ (Fig. 1c). We calculated the ratio of lacZ-positive cells in alb-, Ck19-, α-sma-, or OPN-positive cellsand found the ratio of lacZ + /alb + cells in alb + cells (94.31% ± 2.81%) was significantly higher than that in Ck19 + (1.76% ± 1.25%), α-sma + (0.00% ± 0.00%), or OPN + (0.00% ± 0.00%) cells (Fig. 1d). As a small quantity of Ck19 + cells were reported to also express albumin^10^, lacZ + /Ck19 + cells could be observed. Livers were also double immunostained for lacZ and the progenitor hepatocyte cell marker Axin2. We observed that all Axin2 + cells were lacZ + (Fig. s1d). These results suggest that most of the lacZ was co-expressed with albumin in hepatocytes, but rarely in cholangiocytes, HSCs, or liver stem cells. Thus, the alb + hepatocyte cell lineage tracing system was established *in vivo*.

### Hepatocyte lineage tracing after partial hepatectomy

**Figure 2:**
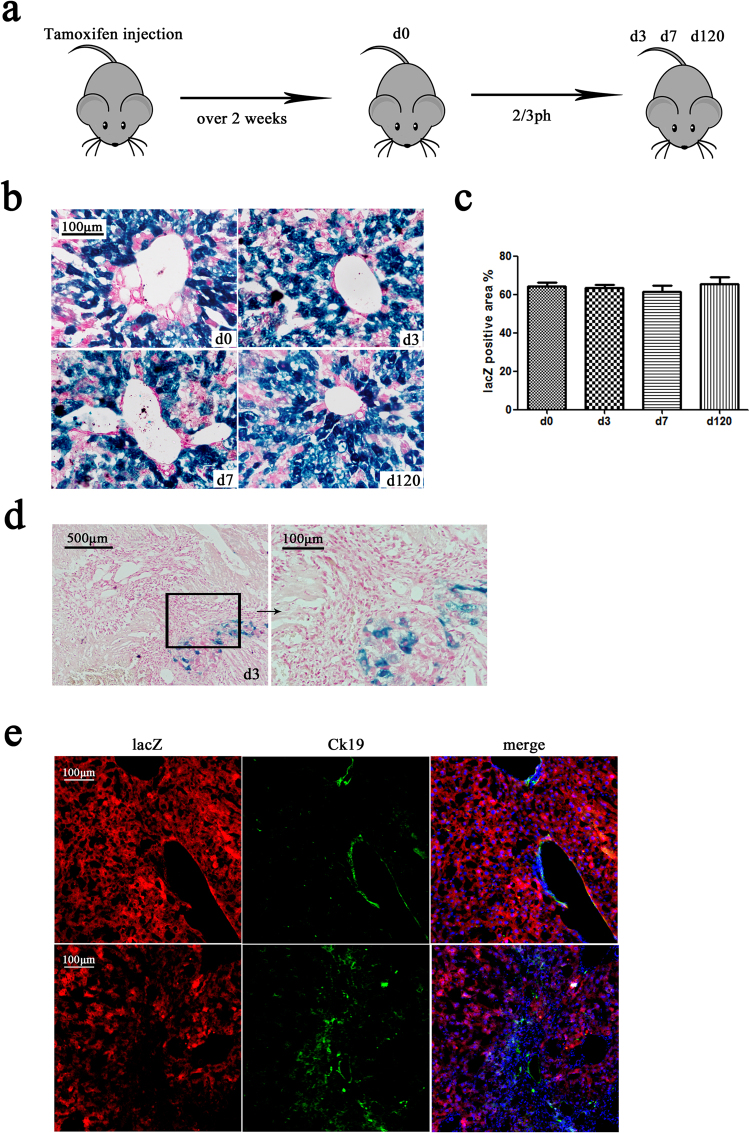
Lineage tracing of hepatocytes after 2/3 partial hepatectomy. (**a**) Experimental scheme: Alb-cre-LacZ mice underwent 2/3 partial hepatectomy after tamoxifen injection. Mice were sacrificed on days 3, 7, and 120. The resected liver lobes were kept as the control (day 0) (n = 3). Magnification, ×400. (**b**) X-gal staining at days 0, 3, 7, and 120. (**c**) Quantification of the X-gal-positive area. The percentage area of lacZ-positive cells on day 0, 3, 7, and 120 were 64.08% ± 9.29%, 63.31% ± 7.53%, 61.40% ± 6.71%, and 65.38% ± 7.98%, respectively. (**d**) X-gal staining of alb-cre-lacZ mice at the wound site on day 3 after 2/3PH. Left line, magnification, ×200; right line, magnification, ×400. (**e**) Double immunostaining for lacZ and Ck19 in the regeneration section (first line) and the wound section (second line) on day 3 after 2/3PH. Nuclei were stained using DAPI (blue). Over 15 sections from a group of mice (n = 3) were counted; data are expressed as the mean ± SEM; magnification, ×200.

To trace the hepatocytes after physical injury, tamoxifen-treated alb-cre-LacZ mice underwent 2/3 partial hepatectomy (PH). The removed liver lobes were used as the control (day 0). Mice were sacrificed on day 3, day 7, and day 120 after partial hepatectomy (n = 3 per group) (Fig. 2a). Following a partial hepatectomy, two types of repair processes commence. The regenerating liver proliferates rapidly for 3 days after a partial hepatectomy. Connective tissue also rapidly grows around the wound. Thus, we investigated the lacZ-labelled hepatocytes in the regenerating liver and in the wound area. In the regenerating liver, the percentage area of lacZ-positive cells on days 0, 3, 7, and 120 were 64.08% ± 9.29%, 63.31% ± 7.53%, 61.40% ± 6.71%, and 65.38% ± 7.98%, respectively (Fig. 2b,c). There was no significant difference between these groups. LacZ-positive cells tended to maintain their overall ratio in the whole liver, indicating that these lacZ-labelled hepatocytes were the main source of progeny hepatocytes in the regenerating liver. At the wound site, many monocytes were observed on day 3 after partial hepatectomy. X-gal stain-positive cells were rarely found among these areas (Fig. 2d). The regenerating livers were double immunostained for Ck19 and lacZ, and the resultsshowed an enrichment of Ck19 + cells in the wound section rather than in the regenerating liver section (Fig. 2e). Most of these Ck19-positive cells were lacZ-negative, which indicated that these cells were not hepatocyte progeny cells. Further, the regenerating livers were doubleimmunostained for ki67 (cell proliferation related marker) and Ck19 or lacZ, and the results showed that both hepatocytes (or hepatocyte-derived cells) and cholangiocytes were proliferating in the regenerating liver (Supplementary Fig. s2). These results indicated that after 2/3 partial hepatectomy injury, the proliferation of hepatocytes and cholangiocytes contributed the most to replacing the lost mass.

### Hepatocyte lineage-tracing in liver homeostasis

**Figure 3:**
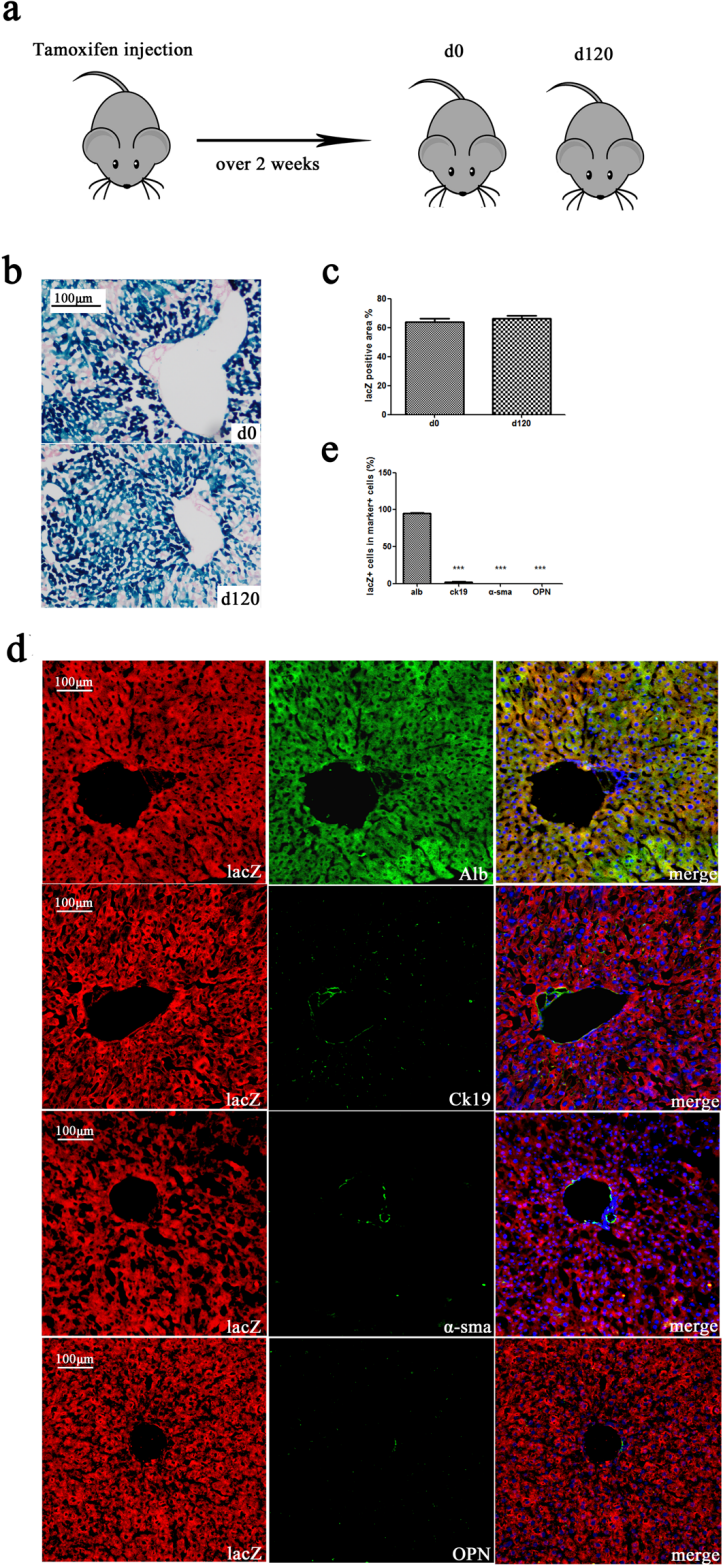
Lineage tracing of hepatocytes in homeostasis. (**a**) Experimental scheme: Alb-cre-lacZ mice were injected with tamoxifen (120 mg/kg) five times every second day for two weeks. Mice were sacrificed at days 0 and 120. (**b**) The ratio of the X-gal-positive area of the liver sections from day 0 (first line) and 120 (second line) was not significantly different. (**c**) Quantification of the X-gal-positive area. The percentage of the lacZ-positive area was 63.93% ± 6.87% and 64.37% ± 3.52% at days 0 and 120, respectively. (**d**) Double immunostaining for lacZ and albumin, Ck19, α-sma, or OPN. (**d**) The ratio of lacZ + /marker + cells in marker + cells. The ratio of lacZ + /alb + cells in alb + cells (95.00% ± 2.30%) was significantly higher than in Ck19 + (1.63% ± 1.42%), α-sma + (0.00% ± 0.00%), or OPN + (0.00% ± 0.00%) cells. Nuclei were stained using DAPI (blue). Over 15 sections from a group of mice (n = 3) were counted; data are expressed as the mean ± SEM; magnification, ×200; ***p < 0.01.

To investigate whether hepatocytes were also the source of progeny hepatocytes during liver homeostasis, mice were treated for 2 weeks with tamoxifen injection (n = 3 per group) and then sacrificed on days 0 and 120 (Fig. 3a). We assessed the ratio of lacZ-positive cells in the liver using X-gal staining. The percentage of the lacZ-positive area was 63.93% ± 6.87% and 64.37% ± 3.52% at days 0 and 120, respectively. There was no significant difference between the two stages (Fig. 3b,c). Liver sections were double immunostained for lacZ and Alb, Ck19, α-sma, or OPN (Fig. 3d). The ratio of lacZ + /alb + cells in alb + cells (95.00% ± 2.30%) was significantly higher than that in Ck19 + (1.63% ± 1.42%), α-sma + (0.00% ± 0.00%), or OPN + (0.00% ± 0.00%) cells. The difference in trend between these groups was the same as on day 0 (Fig. 3e). These results indicated that the progenitor hepatocytes were alb-positive during liver homeostasis. We did not find any obvious transdifferentiation or dedifferentiation in hepatocytes during liver homeostasis.

### Hepatocyte lineage-tracing in a DDC diet injury model

**Figure 4:**
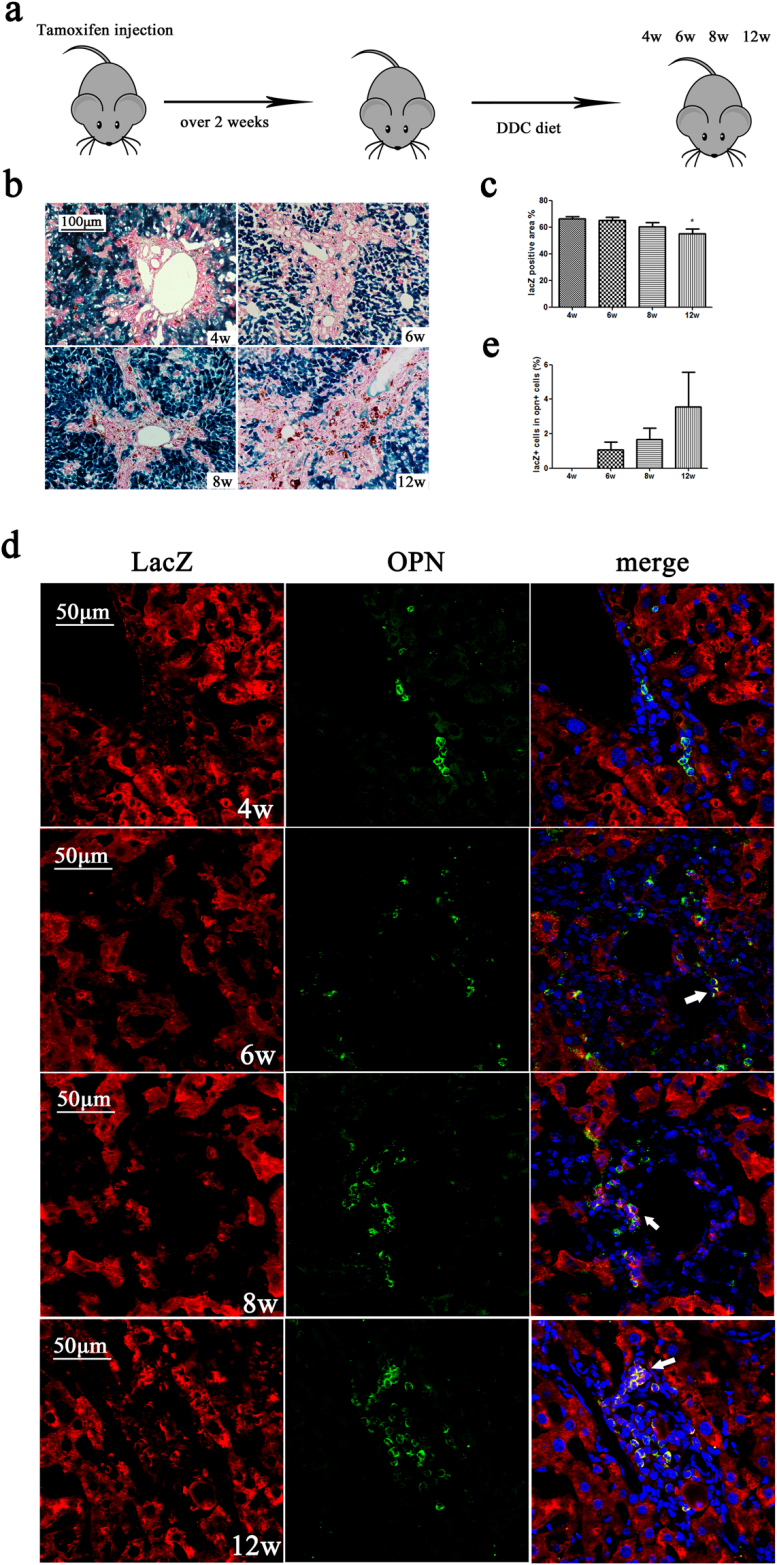
Lineage tracing of hepatocytes in a DDC diet injury model. (**a**) Experimental scheme: Alb-cre-LacZ mice were sacrificed after 4–12 weeks on the DDC diet (n = 3). (**b**) X-gal-staining of liver sections from the 4-, 6-, 8-, and 12-week groups (n = 3). Magnification, ×200. (**c**) Quantification of the X-gal-positive area. (**d**) Double immunostaining for lacZ and OPN at 4–12 weeks. The area indicated by the arrows is enlarged in the top right corner. Magnification, ×600. Nuclei were stained using DAPI (blue). Over 15 sections from a group of mice (n = 3) were counted; data are expressed as the mean ± SEM; *p < 0.05.

The DDC diet injury model is the classical oval cell proliferation model that is widely used in liver regeneration research. We used this model to further study whether hepatocytes have the potential for transdifferentiation or dedifferentiation under non-physiological conditions. Mice were sacrificed after 4–12 weeks on a DDC diet (Fig. 4a). LacZ-negative cells increased during DDC diet injury and the lacZ-positive area decreased (Fig. 4b,c). The liver sections were double immunostained with anti-lacZ and anti-OPN antibodies (Fig. 4d). After a 6-week DDC diet, lacZ/OPN-positive cells could be observed, which suggested the transdifferentiation potential of alb + cells. The liver sections were double stained with anti-lacZ and anti-alb antibodies (Supplementary Fig. s3a). The ratio of lacZ + /alb + cells in alb + cells did not change in response to DDC diet injury (Fig. s3b,c). These results indicated that the alb + hepatocytes were still mainly derived from the original lacZ-labelled cells.

### Hepatocyte lineage-tracing in a CCl4 injury model

**Figure 5:**
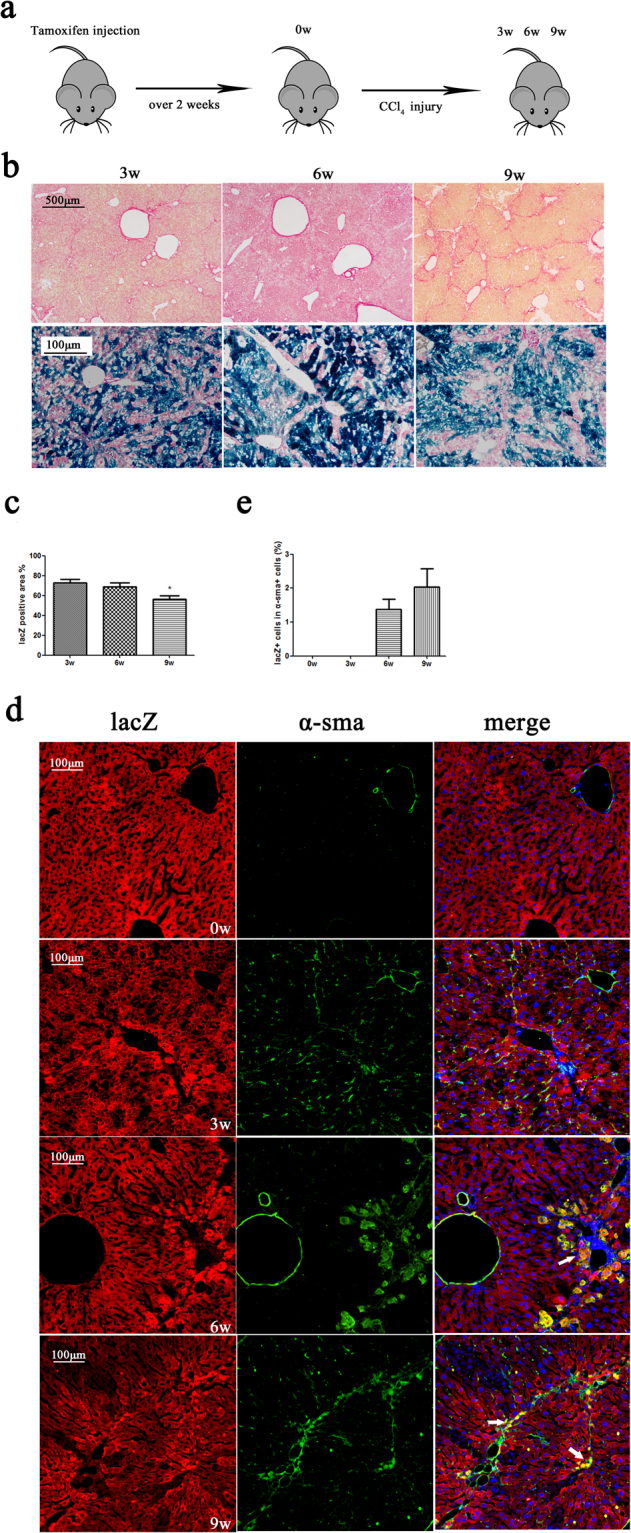
Lineage tracing of hepatocytes following CCl4 injury. (**a**) Experimental scheme: Alb-cre-LacZ mice were sacrificed after 3–9 weeks of CCl_4_ treatment (n = 3). (**b**) Sirius staining (first line) of the liver sections fromthe 3- to 9-week groups (n = 3); magnification, ×40 and X-gal-staining (second line) of the liver sections from the 3- to 9-week groups (n = 3); magnification, ×200. (**c**) Quantification of the X-gal-positive area. (**d**) Double immunostaining for lacZ (red) and α-sma (green) after 3–9 weeks of CCl_4_ treatment. Arrows point to lacZ + /α-sma + cells. Magnification, ×600. Nuclei were stained using DAPI (blue). Over 15 sections from a group of mice (n = 3) were counted; data are expressed as the mean ± SEM; *p < 0.05.

Mice were injected with CCl4 (1 mL/kg diluted in corn oil) twice per week and then sacrificed at 3, 6, and 9 weeks (Fig. 5a). The Sirius-stain-positive area increased, while the X-gal-positive area decreased at 3, 6, and 9 weeks (Fig. 5b). More LacZ-negative cells were observed during CCl_4_ injury (Fig. 5c). Hepatocytes and hepatocyte-derived cells were labelled with lacZ, while HSCs were labelled with α-sma. LacZ + /α-sma + cells appeared after 6 weeks of CCl_4_ treatment (Fig. 5d, Supplementary Fig. s4a). Snail was used as a potential marker of epithelial-to-mesenchymal transition as it was reported to play an important role in EMT^14^. Snail + and lacZ + cells were observed after 6–9 weeks of CCl_4_ treatment (Supplementary Fig. s4b). The ratio of lacZ + /alb + cells in alb + cells did not change during CCl_4_ injury, which suggested that the hepatocytes mainly originated from alb + cells (Supplementary Fig. s4c,d). It is worth noting that OPN + /lacZ + cells were observed after 6 weeks of CCl_4_ treatment (Supplementary Fig. s4e). This suggests that hepatocytes may have the potential for transdifferentiation. We also observed the same situation after bile duct ligation (BDL) injury. Two weeks after BDL injury, a small quantity of lacZ + /α-sma + cells were detected, and Snail + and lacZ + cells were also observed (Supplementary Fig. s5a,b). These results suggest that some of the α-sma-positive proliferating cells were the progeny of lacZ-labelled cells. These dual positive cells may support the hypothesis that hepatocytes undergo EMT in liver fibrosis but that their quantity is small.

## Discussion

In this paper, we studied the cell fate of hepatocytes during normal liver homeostasis and in several pathological liver regeneration models. Albumin was used as the hepatocyte cell marker, and the CreERT/lacZ-based lineage tracing method was used. Compared with Ttr, Tbg, or Axin2^15–17^, albumin-positive cells might contain more types of hepatocytes including Axin2 + cells (Fig. s1c). Therefore, different types of hepatocytes were included in our observations. Under liver homeostasis conditions and all liver injury models studied, our results indicated that alb + hepatocytes were the main source of progeny hepatocytes. Wang, B. *et al*. found that Axin2 + cells were the source of new hepatocytes during liver homeostatic renewal^15^. This conclusion was not inconsistent with our result because Axin2 + cells were also alb positive.

Our results in the 2/3 partial hepatectomy liver injury model were consistent with recent research, which showed that in the regenerated liver, mature hepatocytes were the source of progeny hepatocytes^10^. However, many cells at the wound-healing site were not from the mature hepatocytes. These cells were Ck19 + /lacZ- cells, which might be progeny of cholangiocytes or oval cells. Rapid proliferation of Ck19 + cells in the wound area was not observed in the regenerating liver. These results suggest that cholangiocytes and hepatocytes have different functions in liver regeneration after 2/3 partial hepatectomy injury. We did not observe noticeable dedifferentiation or transdifferentiation of hepatocytes during normal liver homeostasis and in the 2/3 partial hepatectomy liver regeneration model.

In the DDC diet model, proliferation of oval cells, which are considered to be liver stem cells, is enhanced. Tarlow, B. D. *et al*. found that hepatocytes contribute to the progenitor pool after 6 weeks in the liver injury model^18^. Our results are consistent with their reports, for lacZ/OPN-positive cells were observed in mice on DDC diet. However, it is worth noting that lacZ + /Ck19 + cells may also be involved in this process.

A small amount of lacZ + /α-sma + double-positive and lacZ + / snail + double-positive cells appeared in the liver after CCl_4_ or BDL treatment. In previous reports, whether hepatocytes underwent EMT was contentious. The experiment results *in vitro* suggested that hepatocytes could dedifferentiate via EMT while cultured in the absence of added growth factors or when induced by TGF-β1^12,19^. Zeisberg, M. *et al*. used AlbCre. R26RstoplacZ double-transgenic mice to demonstrate that hepatocytes that undergo EMT contribute substantially to the population of FSP1-positive fibroblasts in CCl_4_-induced liver fibrosis^20^. However, it has been argued that FSP1 is not a reliable marker used to define fibrosis^21^. Therefore, these conclusions may have been inaccurate. Meanwhile, Taura, K. *et al*. found that hepatocytes do not undergo EMT in liver fibrosis by using triple transgenic mice expressing Rosa26 stop β-gal, albumin Cre, and collagen α1 GFP. However, exclusive reliance on a reporter mouse system might resultin potentially missing collagen-producing mesenchymal cells^13^. Another reason for the different results might be the degree of liver fibrosis. Our results are consistent in that no lacZ + /α-sma + cells were observed within 3 weeks of CCl_4_ injection, but after 6 weeks of CCl_4_ treatment, lacZ + /α-sma + cells were observed. In the BDL injury model, lacZ + /α-sma + cells were also observed after 2 weeks of injury. These results support the hypothesis that hepatocytes might undergo EMT during the process of liver fibrosis. However, it has been reported that transplanted haematopoietic stem cells (HSCs) or macrophages can fuse with the host hepatocytes *in vivo* ^22^. Therefore, cell fusion provides another potential source of double positive cells, and whether it occurs in a non-transplant model should be further studied.

In conclusion, in this study, we observed the appearance of lacZ/OPN-positive and lacZ/α-sma-positive cells, which may be derived from hepatocytes in the DDC diet and CCl_4_ injury model, respectively. These results indicate the potential of hepatocytes to undergo transdifferentiation or dedifferentiation in response to DDC diet injury and EMT processes during liver fibrosis. Although we do not know whether hepatocyte EMT is a result of fibrogenesis or vice versa, this phenomenon may explain the strong relationship between liver cancer and hepatocirrhosis. In fact, a recent study suggested that hepatocellular carcinoma originates from hepatocytes^16^. Additionally, in liver fibrosis injury models, a small amount of lacZ/OPN-positive cells were also observed (Supplementary Figs s4e and s5c). The significance and functions of these hepatocyte-derived cells *in vivo* should be further investigated.

## Methods

### Mice

Albumin-promoted CreERT knock-in (Alb-KI) heterozygotic mice and Rosa26-LSL-LacZ mice on the C57BL/6 strain background were housed under specific pathogen-free conditions and maintained under controlled conditions (21 °C–24 °C; 12-h light-dark periods). Alb-KI mice were mated with Rosa26-LSL-lacZ mice to produce Cre/lacZ double-positive mice (Alb-cre-lacZ). Alb-cre-lacZ mice (8–10 weeks old, male) were used in the experiments. All animals were provided by Shanghai Biomodel Organism Science and Technology Development Co., Ltd. (Shanghai, China).

Animal experiments in this study were approved by the Institutional Animal Care and Use Committee at the Shanghai Research Centre for Model Organisms (SRCMO) (IACUC No. 2012-0003-01) and conducted in accordance with government guidelines for animal care.

### Tamoxifen-induced cre expression

Tamoxifen (120 mg/kg) diluted in corn oil (both from Sigma-Aldrich, USA) was intravenously injected into Alb-cre-lacZ mice for 10 days every ofther day. The mice were then housed for at least 2 weeks to wash out any remaining tamoxifen.

### X-gal staining

Livers were perfused with cold 4% paraformaldehyde/PBS and post-fixed for 4 h. Liver samples were dehydrated in 15% sucrose (Sigma-Aldrich, USA) overnight at 4 °C and transferred to 30% sucrose for 8 h, then embedded into an optimal cutting temperature compound (Thermo, USA) for cryopreservation or for cryosectioning into 8- to 10-μm sections. Frozen sections were stained overnight using X-gal (Beyotime Biotechnology, Shanghai, China)^23^. The nuclei were stained using nuclear fast red (Sigma-Aldrich, USA). Images were measured using Image-pro software.

### Immunostaining

Tissue samples were perfused with cold 4% paraformaldehyde/PBS and fixed overnight, dehydrated in 15% sucrose (Sangon, Shanghai, China) for 8 h and 30% sucrose overnight at 4 °C, embedded in OCT, and cut into 10-μm-thick sections. Sections were blocked in Immunol Staining Blocking Buffer (Beyotime Biotechnology, Shanghai, China) for 1 h, and then incubated with primary antibodies (Supplemental Table 1) overnight at 4 °C and with secondary antibodies (Supplemental Table 2) for 1 h at room temperature. Nuclear DNA was stained using 4′,6-diamidino-2-phenylindole (DAPI) (Invitrogen, USA).

### 2/3 Partial hepatectomy

Ketamine (JiangsuHengrui Medicine Co., LTD, Lianyungang, China) (100 mg/kg) and Xylazine (Sigma-Aldrich, USA) (10 mg/kg) was used as an anesthetic and diluted in physiological saline. The left lateral lobe and the middle lobe of the liver were surgically removed.

### 3,5-diethoxycarbonyl-1,4-dihdrocollidine-containing diet

Mice received a diet containing 0.1% w/w DDC (3,5-diethoxycarbonyl-1,4-dihdrocollidine; Sigma-Aldrich, USA) for 4–12 weeks^24^.

### Carbon tetrachloride chronic intoxication

CCl_4_ (1 µL/g body weight), diluted to 20% in corn oil (both from Sigma-Aldrich,USA), was injected twice per week for 3–9 weeks.

### Microscope image acquisition and quantification

X-gal-stained sections were imaged using a Nikon Microscope ECLIPSE 90i (Nikon, USA). Co-localization images were obtained using Olympus Fv10i confocal microscopy (Olympus, Japan). For X-gal-stained area quantification, images were analyzed using Image-Pro Plus 6.0. For fluorescence co-localization statistics, images were acquired and background corrected using FV10-ASW software^25^.

### Statistics

Data are expressed as the mean ± standard error of the mean. Statistical significance was determined using a one-way analysis of variance. P < 0.05 was considered significant. Samples were obtained from three animals per group. Immunostaining image statistics were acquired and analyzed from three different sections per sample (n = 3 per group) and over five visual fields (magnification, × 400) per section containing a portal vein or central vein.

## Electronic supplementary material

Supplementary information
