# Supplementary material for: Fate tracing of hepatocytes in mouse liver
Source: Sci Rep. 2017 Nov 23;7:16108. doi: 10.1038/s41598-017-15973-7 (PMC5701080; doi:10.1038/s41598-017-15973-7)
Supplement: Supplementary file 1 — Supplementary information [file 41598_2017_15973_MOESM1_ESM.doc]

**Fate tracing of hepatocytes in mouse liver**

Xiaowen Gu^1^, Danyi Huang^1^, Lei Ci^1^, Jiahao Shi^1^, Mengjie Zhang^1^, Hua Yang^1^, Zhugang Wang^2^, Zhejin Sheng^1^, Ruilin Sun^2^& Jian Fei^1,2^*

1 School of Life Science and Techonology, Tongji University. Shanghai 200092, China.

2 Shanghai Engineering Research Center for Model Organisms, SRCMO/SMOC. Shanghai 201203, China.

**Supplementary Figure s1**

| a |
| --- |
| 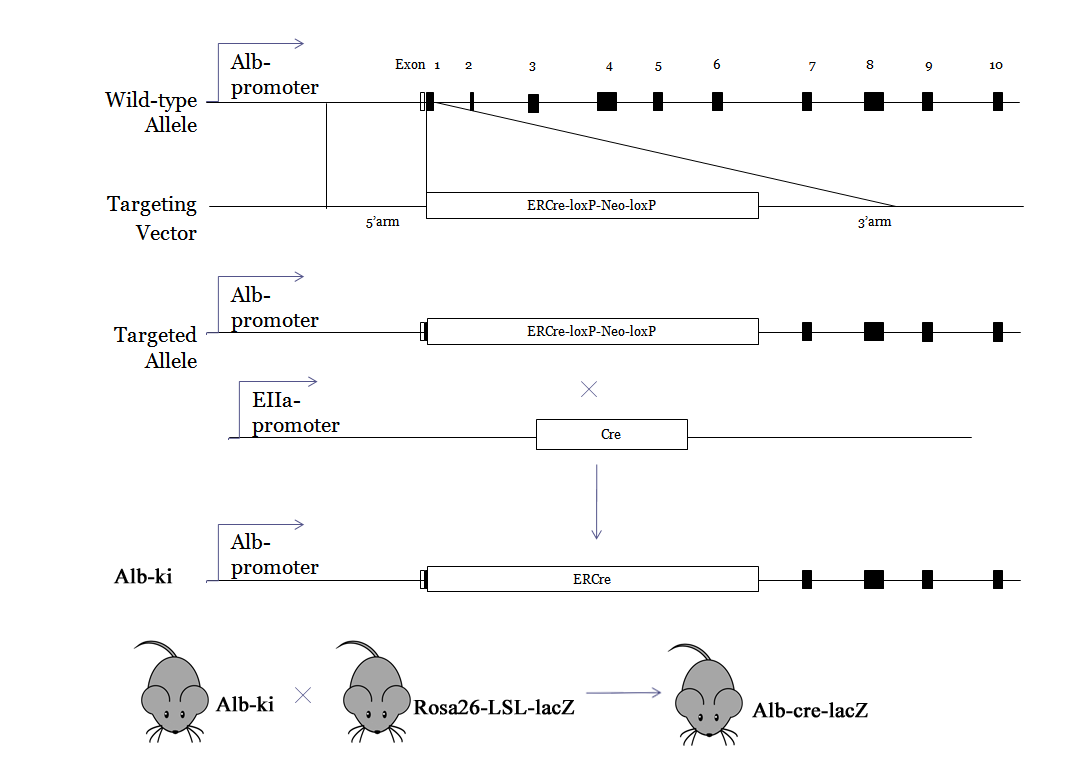   | b | c | | --- | --- | |  |  | |
| d  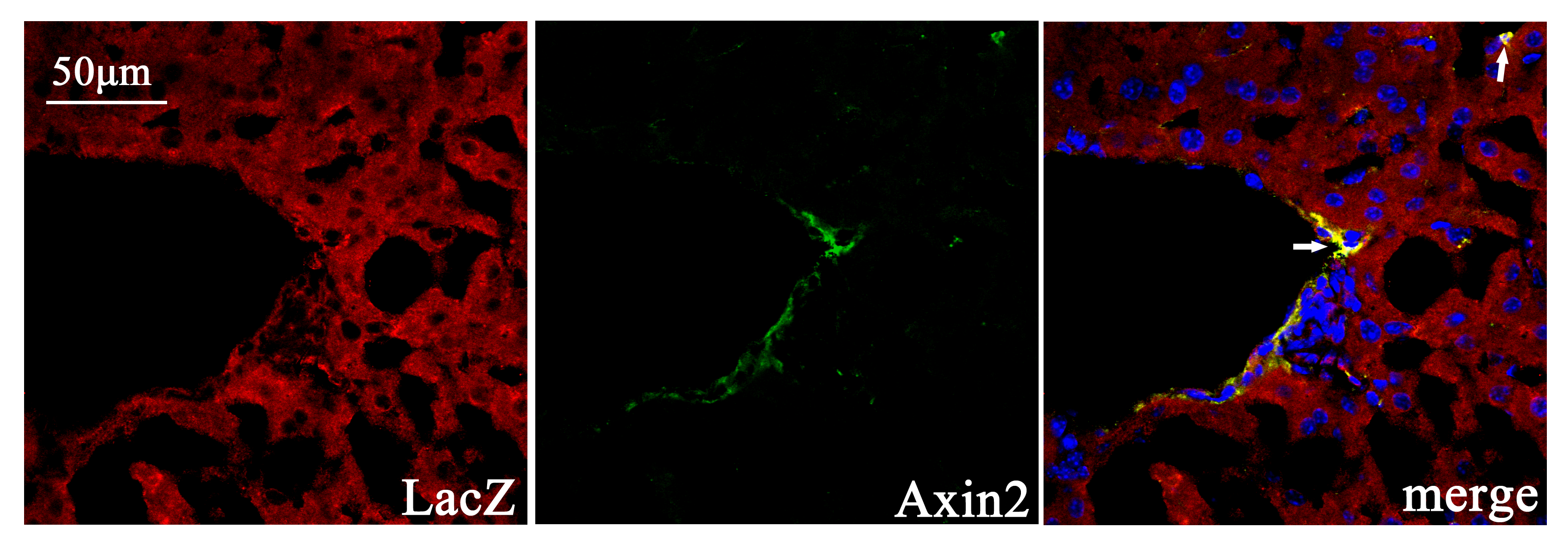 |

Supplemental Figure 1. Construction of alb-ki-lacZ mice. (a) Construction of alb-ki-lacZ mice. (b) Liver function test results. ALB: albumin; ALT: alanine aminotransferase; TP: total protein. (c) Quantification of the X-gal-positive area. (d) Co-immunostaining for lacZ (red) and the progenitor hepatocyte marker Axin2 (green). Arrows point to lacZ/Axin2 positive cells. Nuclei were stained using DAPI (blue), Over 15 sections from a group of mice (n=3) were counted; magnification, ×600; *p<0.05

**Supplementary Figure s2**


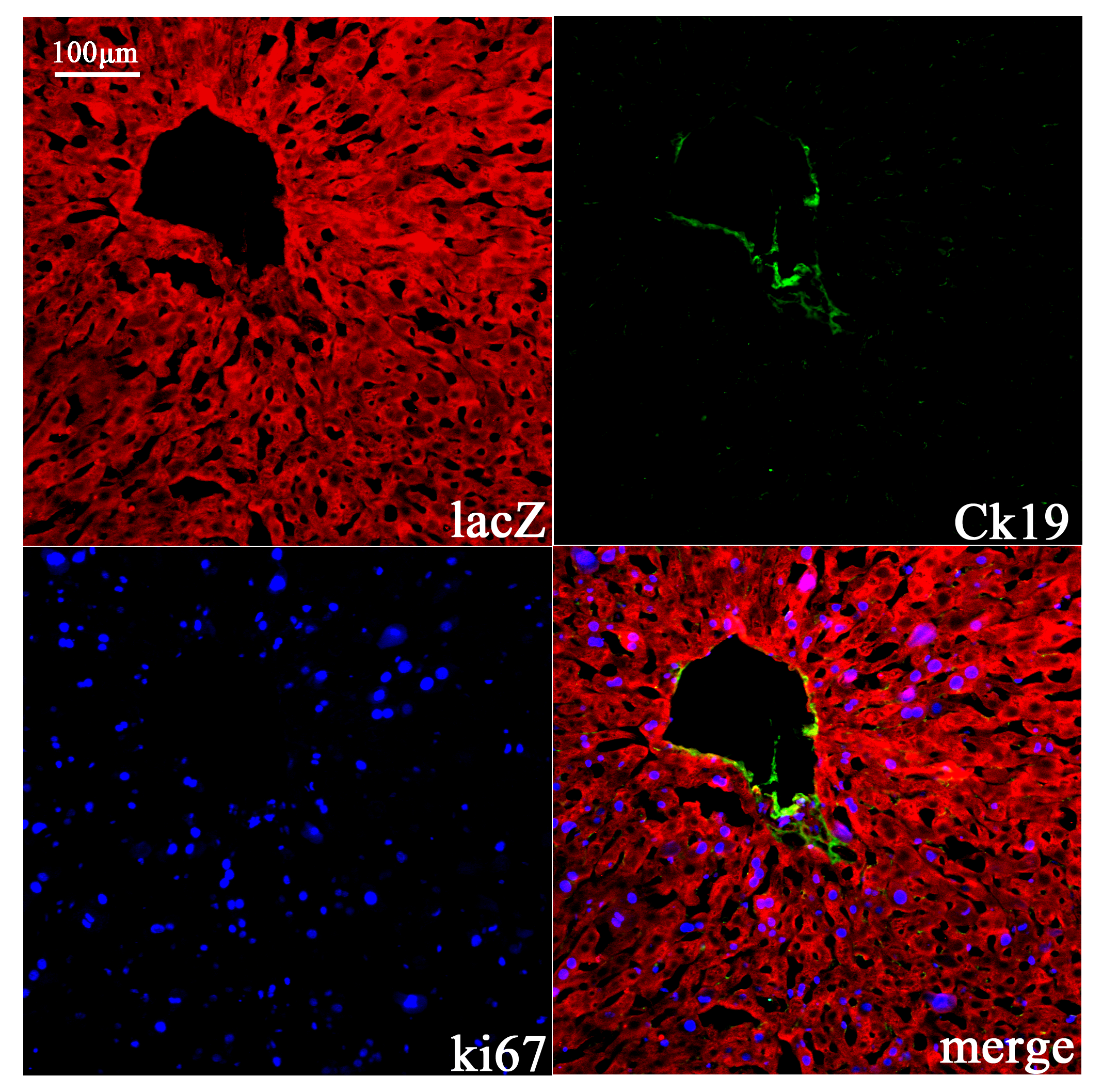


Supplemental Figure 2. Cell proliferation in the regenerating liver after 2/3 partial hepatectomy liver injury. Co-immunostaining for lacZ (red), ck19 (green) and ki67 (blue) in the regeneration section. Over 15 sections from a group of mice (n=3) were observed; magnification, ×200.

**Supplementary Figure s3**

| a | |
| --- | --- |
| 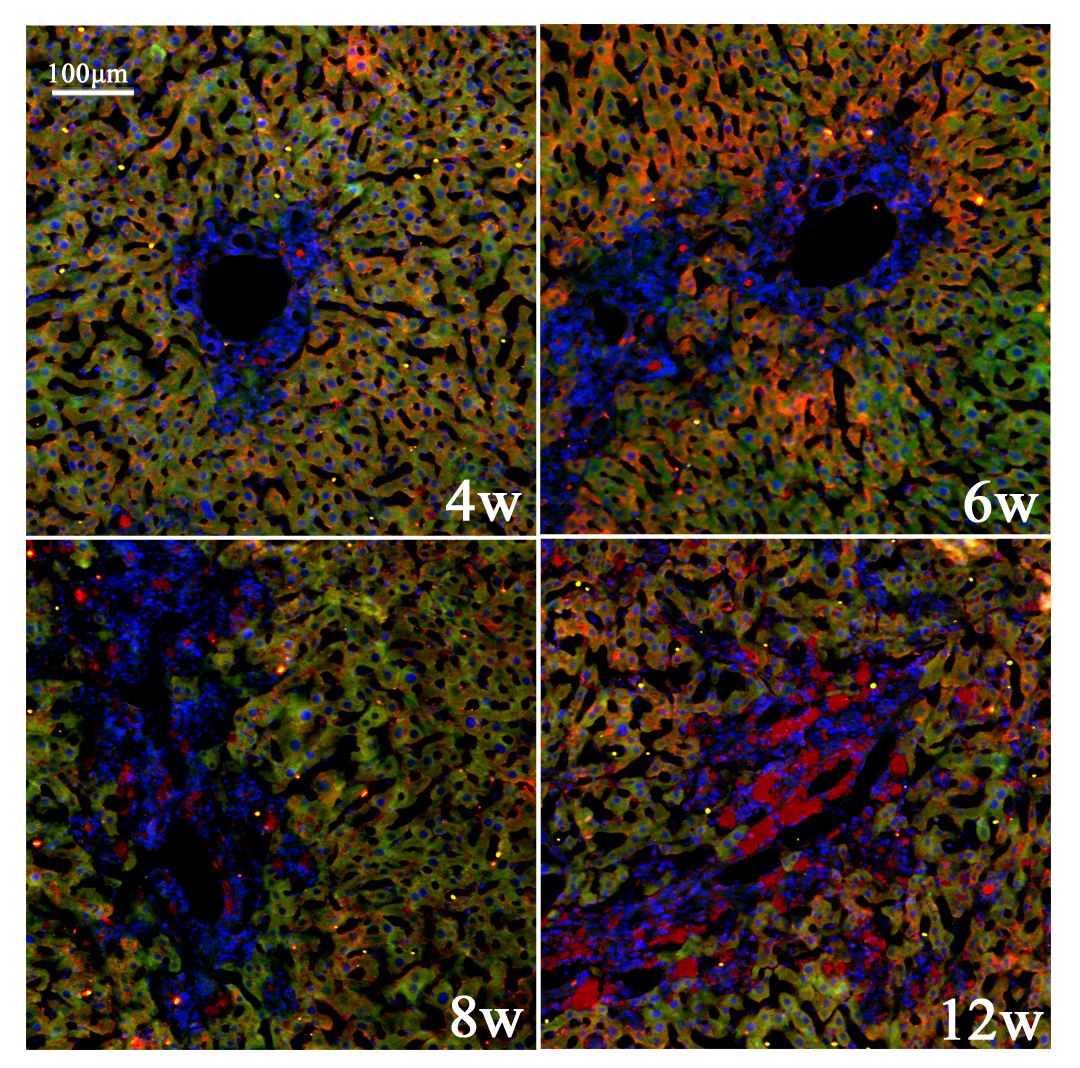 | |
| b | c |
|  |  |

Supplemental Figure 3. Co-localization of alb and lacZ on the DDC diet. (a) Co-immunostaining for lacZ (red) and alb (green) after 4–12 weeks on the DDC diet. Nuclei were stained using DAPI (blue). magnification, ×200. (b) Quantification of the ratio of lacZ+/alb+ cells in alb+ cells. (c) The Pearson’s coefficient of lacZ+ and alb+. Over 15 sections from a group of mice (n=3) were observed.

**Supplementary Figure s4**

a


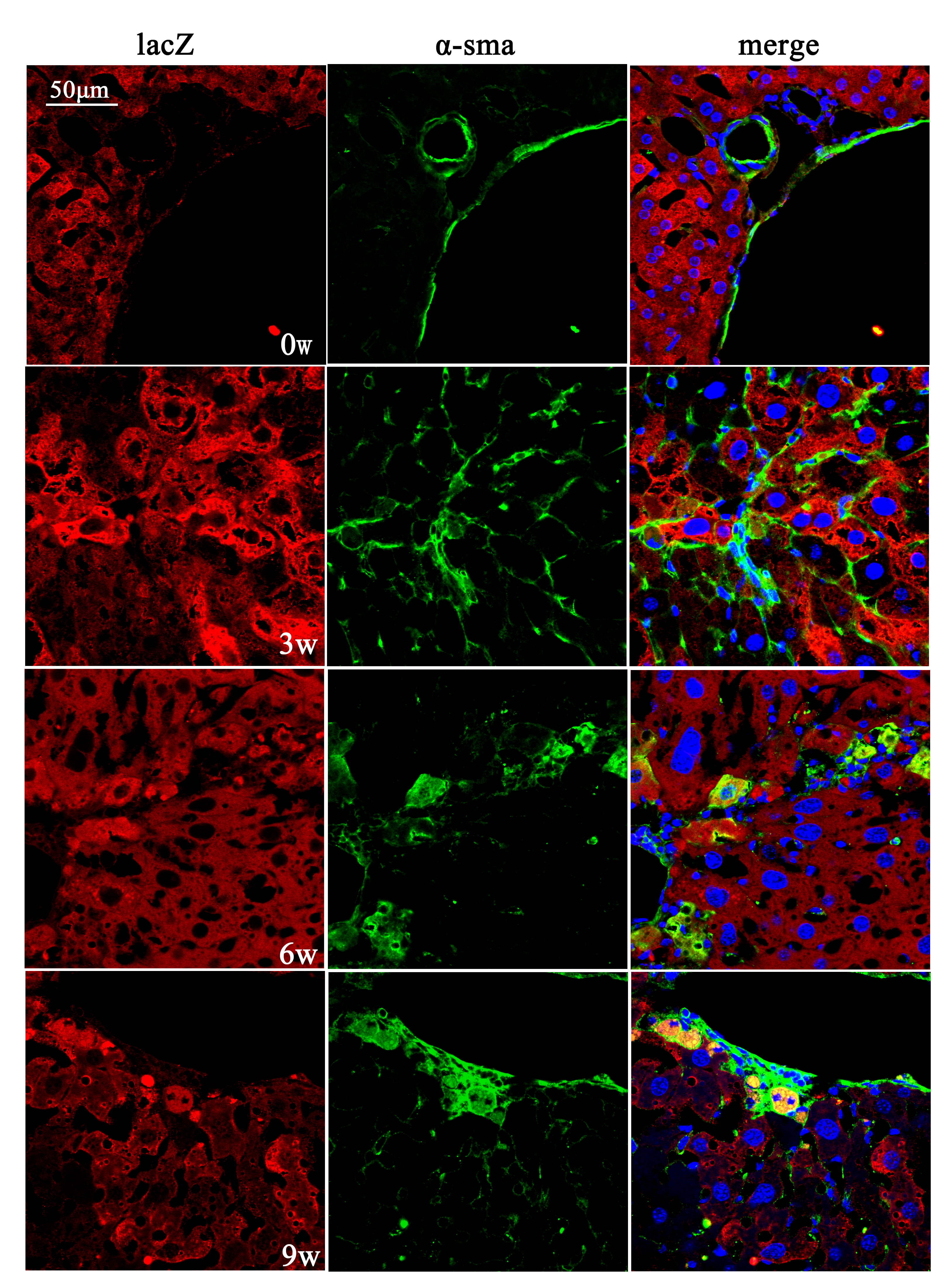


| b |
| --- |
| 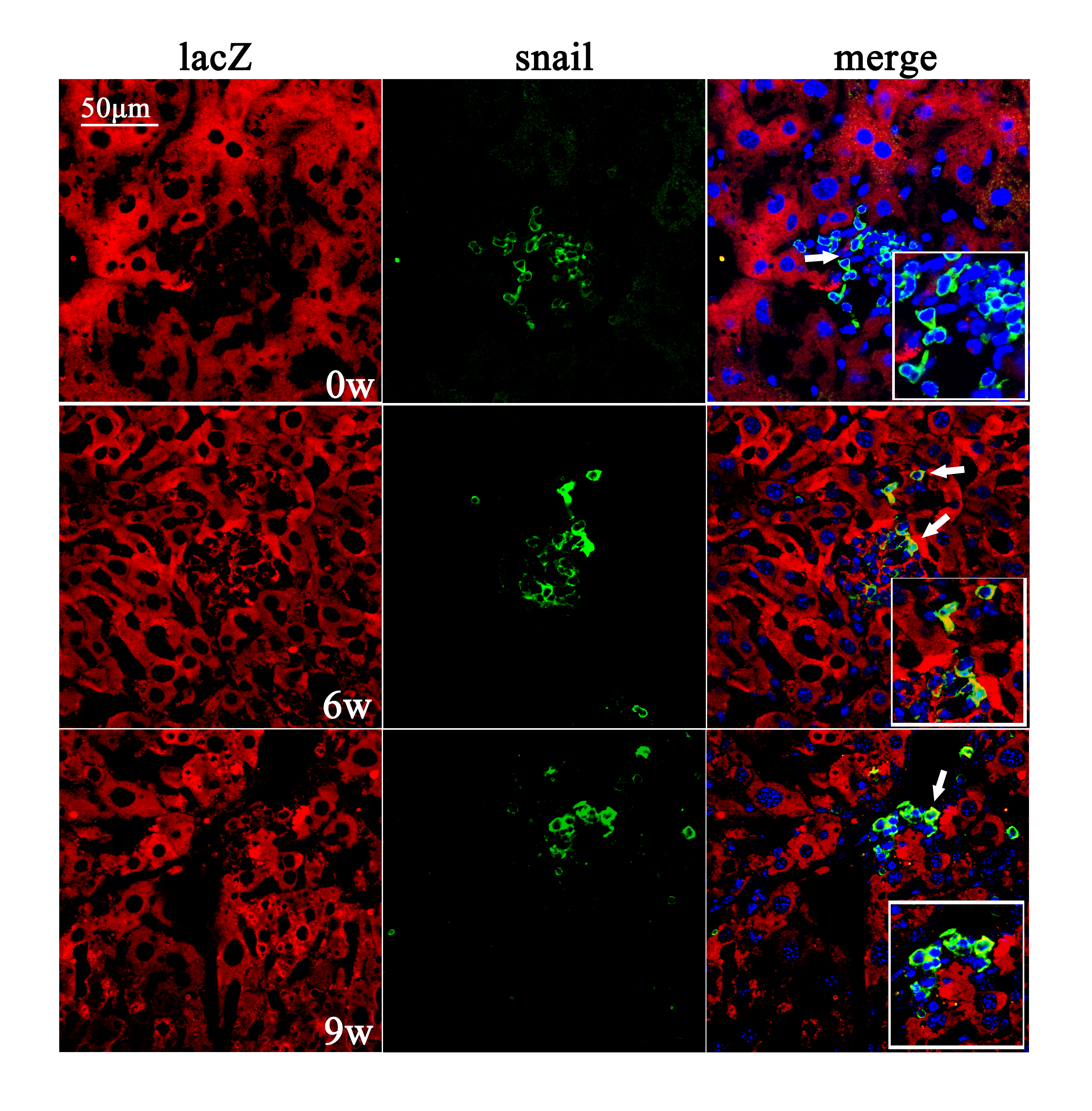 |
| c |
| 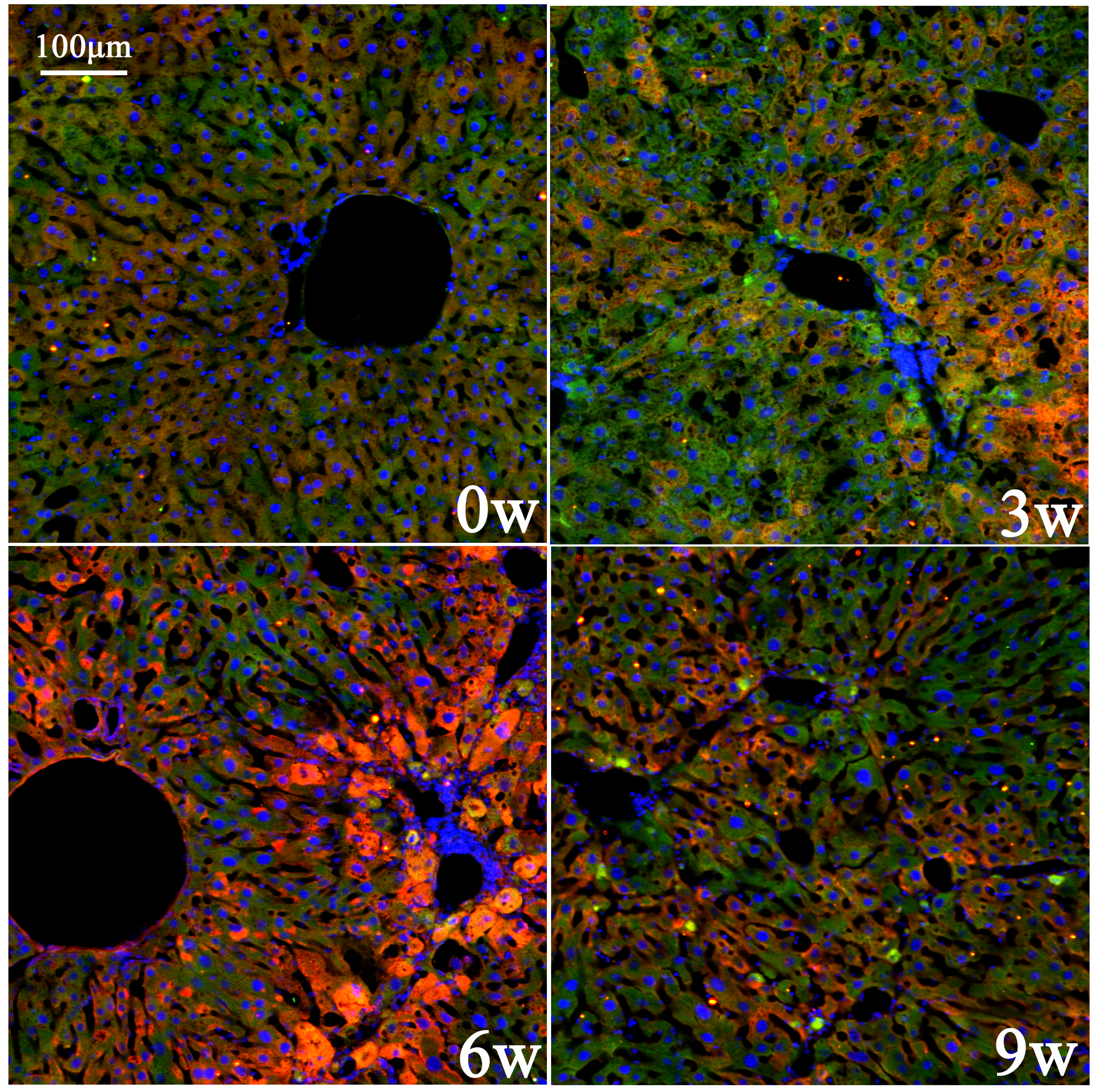 |
| d |
|  |
| e |


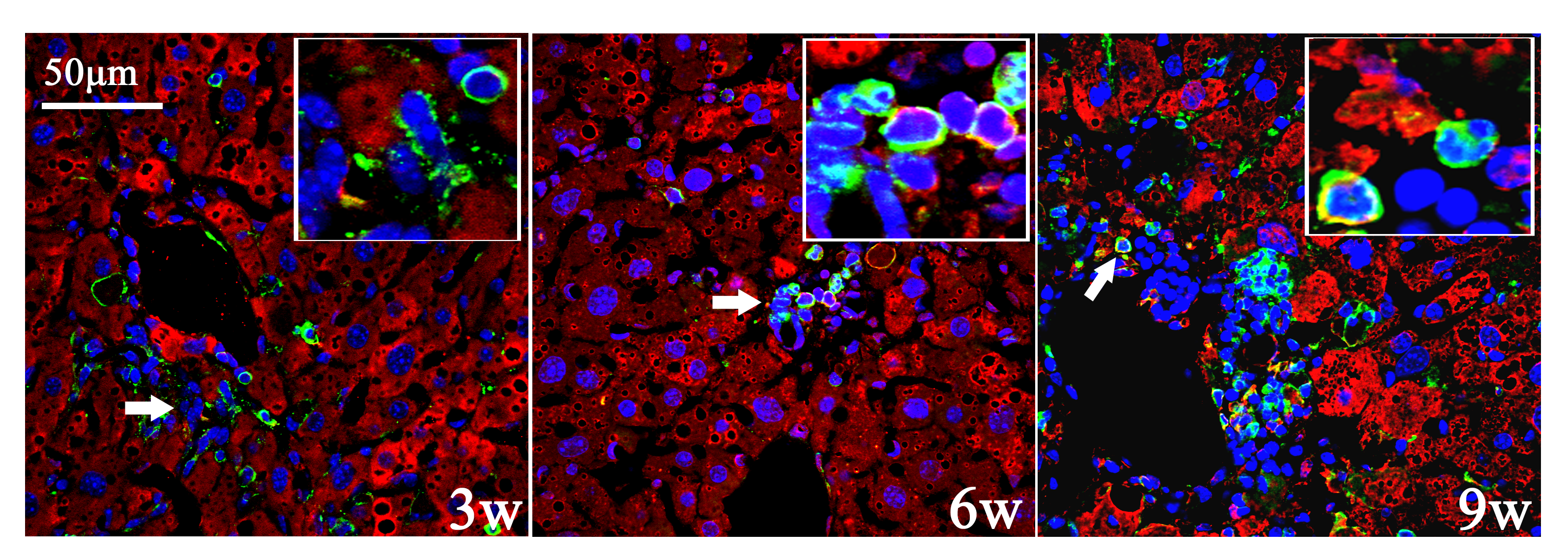


Supplemental figure 4 hepatocytes tracing in ccl4 injury. (a) Co-immunostaining for lacZ(red), α-sma (green) after 3-9 weeks in ccl4 chronic liver injury. Nuclei were stained using DAPI (blue), magnification, 600x. (b) Co-immunostaining for lacZ(red), SNAIL + SLUG (green) in 6-9 weeks ccl4 chronic liver injury. Nuclei were stained using DAPI (blue), magnification, 600x. (c) Co-immunostaining for lacZ(red), alb (green) in 3-9 weeks ccl4 chronic liver injury. Nuclei were stained using DAPI (blue), magnification, 200x. (d) Qualification of the ratio of lacZ^+^/alb^+^ cells in alb^+^ cells and the Pearson’ Coeff. of lacZ^+^ and alb^+^ cells. *p<0.05. Over 6 sections from a group of mice (n=3) were observed; magnification, 200x. (e) Co-immunostaining for lacZ (red), OPN (green) in 3-9 weeks ccl4 chronic liver injury. The area pointing by the arrows was enlarged on the top right corner. Nuclei were stained using DAPI (blue)

**Supplementary Figure s5**

| a | |  |
| --- | --- | --- |
| 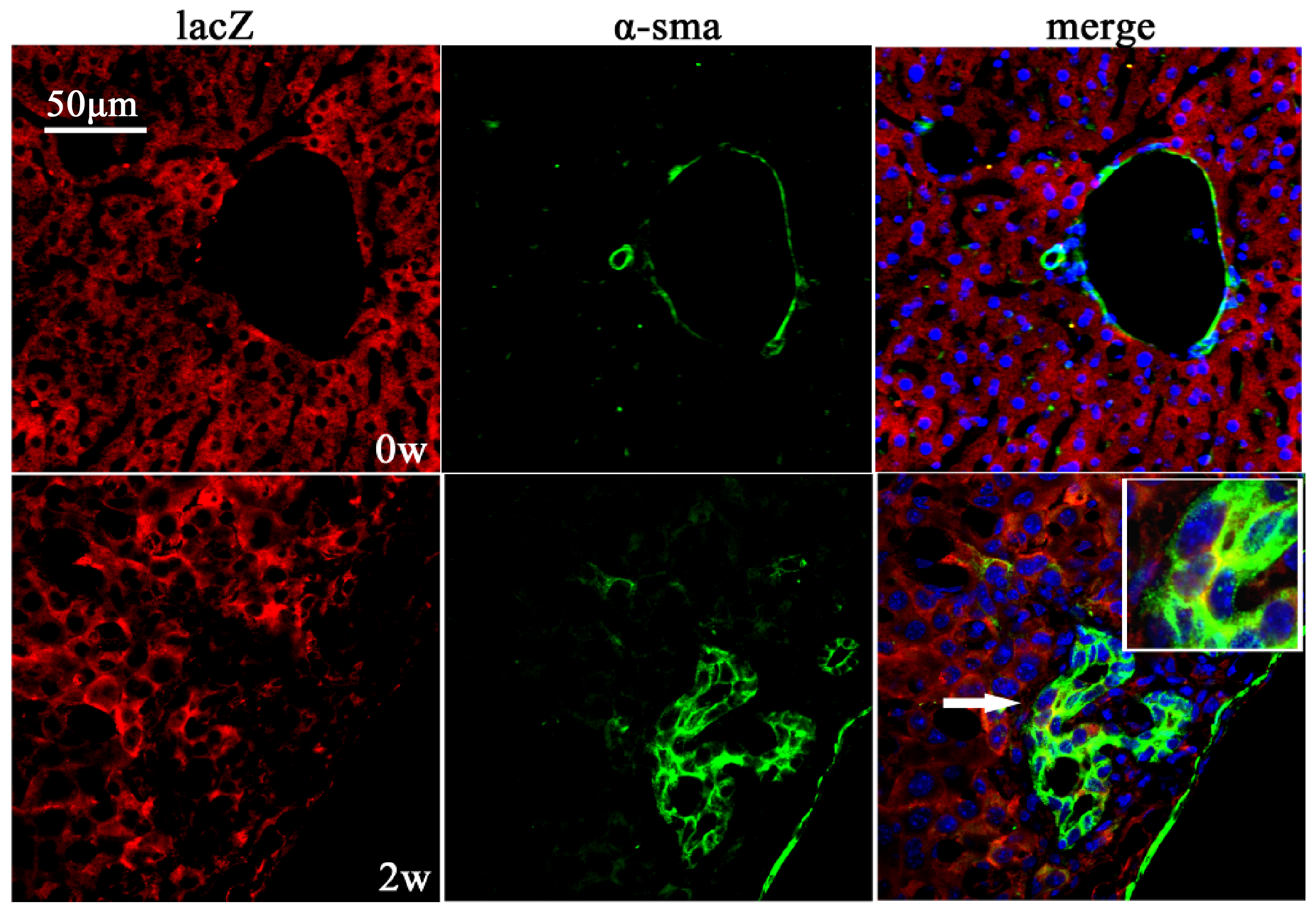 | | |
| b |  | |
| 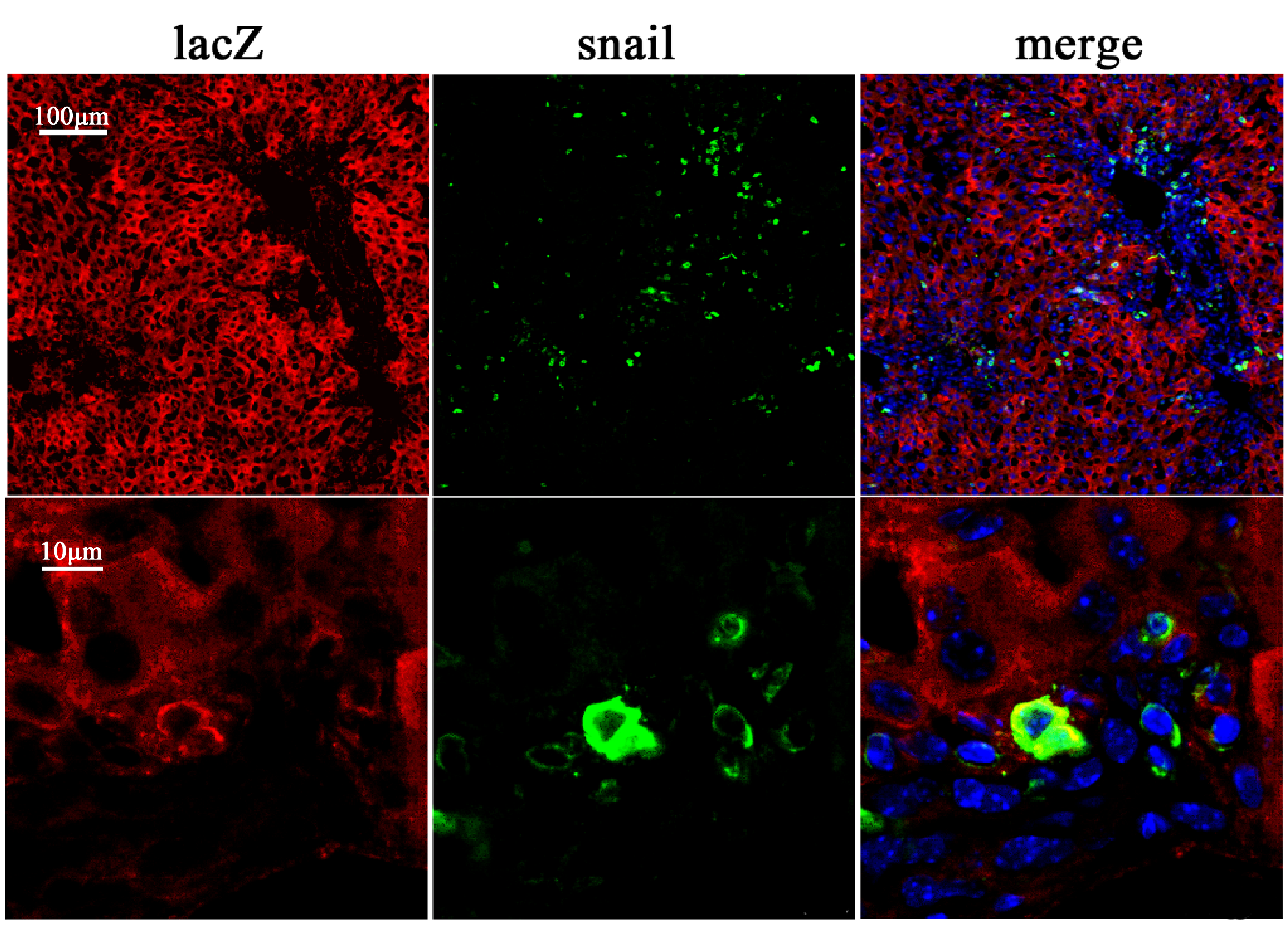 | | |
| c |  | |
| 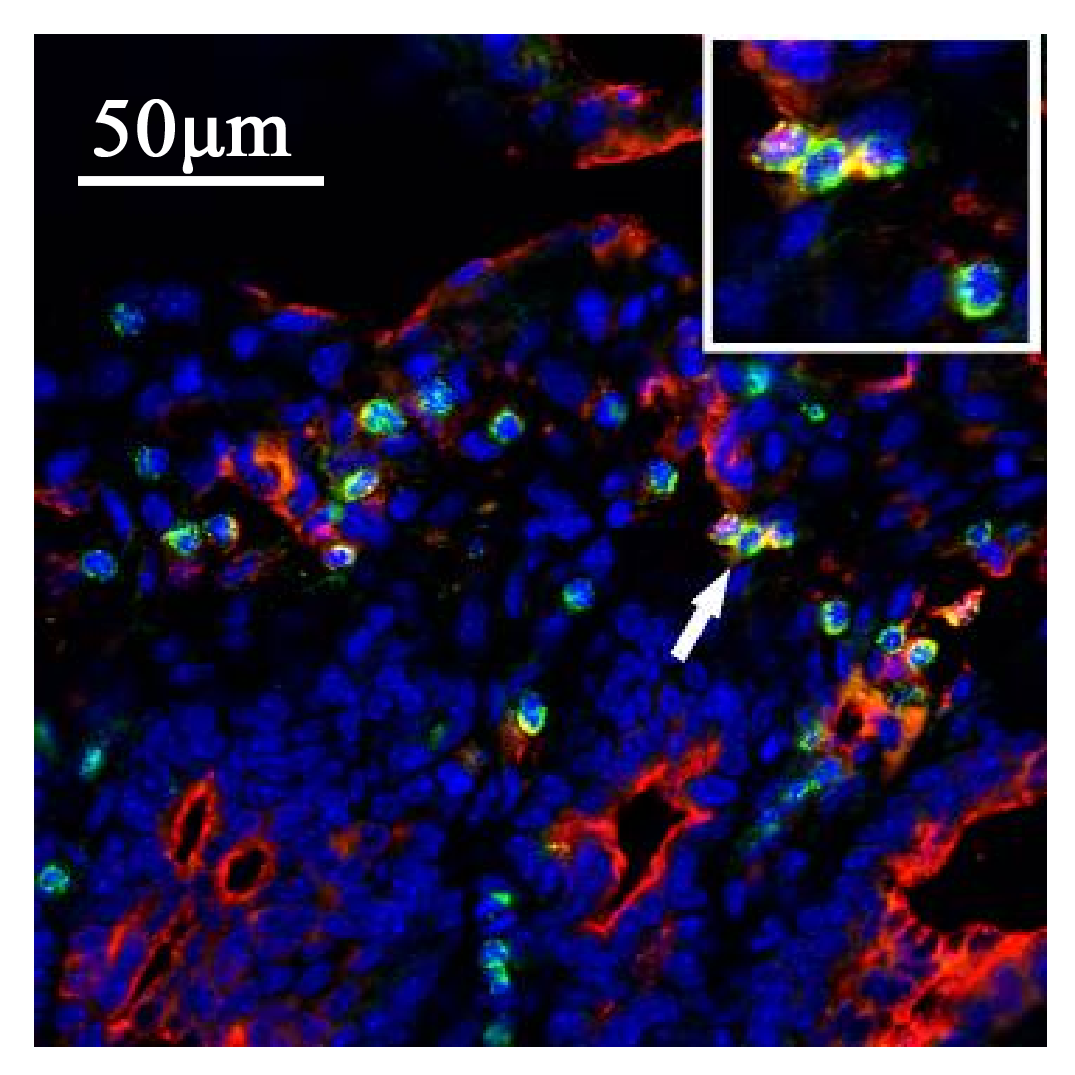 |  | |

Supplemental figure 5 hepatocytes tracing in BDL injury. (a) Co-immunostaining for lacZ (red), α-sma (green) after 2 weeks in BDL liver injury. Arrows pointing to lacZ+/α-sma+ cells. magnification, 600x. Nuclei were stained using DAPI (blue) (b) Co-immunostaining for lacZ(red), SNAIL + SLUG (green) after 2 weeks BDL liver injury. first panel magnification, 100x; second panel magnification,1800x. Nuclei were stained using DAPI (blue) (c) Co-immunostaining for lacZ(red), OPN(green) after 2 weeks BDL liver injury. magnification, 200x Nuclei were stained using DAPI (blue). The area pointing by the arrows was enlarged on the top right corner. The area pointing by the arrows was enlarged on the top right corner.

**Supplemental Tables**

Supplementary Table1. details of primary antibodies

| antibody | manufacturer | Catalog number |
| --- | --- | --- |
| Sheep Anti-Human Serum Albumin | Abcam (UK) | ab8940 |
| Chicken Anti-beta Galactosidase | Abcam (UK) | ab9361 |
| Rabbit Anti-alpha smooth muscle Actin | Abcam (UK) | ab32575 |
| Mouse Anti-Cytokeratin 19 Antibody | Millipore (USA) | mab3238 |
| Rabbit Anti-Axin2 antibody | Abcam (UK) | ab109307 |
| Rabbit Anti-SNAIL + SLUG antibody | Abcam (UK) | ab180714 |
| Rabbit Anti-Ki67 | Abcam (UK) | ab15580 |
| Rabbit Anti-Osteopontin | Abcam (UK) | Ab91655 |

Supplementary Table2. detail of secondary antibodies

| antibody | manufacturer | Catalog number |
| --- | --- | --- |
| Goat Anti-Chicken IgY H&L (Cy3) | Abcam (UK) | ab97145 |
| donkey anti-sheep IgG-FITC | SantaCruZ (USA) | sc-2476 |
| Goat anti-rabbit (Alexa Fluor 647) | Beyotime (China) | A0473 |
| Goat anti-rabbit (FITC) | Beyotime (China) | A0562 |
| Goat anti-mouse (FITC) | Beyotime (China) | A0568 |
